# Time to consider catheter ablation as an alternative to implantable cardioverter-defibrillator therapy in high-risk patients with Brugada syndrome?

**DOI:** 10.1093/europace/euad338

**Published:** 2023-11-09

**Authors:** Giulio Conte, Vincent Probst

**Affiliations:** Division of Cardiology, Cardiocentro Ticino Institute, Ente Ospedaliero Cantonale, Via Tesserete 48, Lugano CH-6900, Switzerland; Faculty of Biomedical Sciences, Università della Svizzera Italiana, Lugano, Switzerland (USI); Service de Cardiologie, L'institut du thorax, CHU Nantes, Nantes, France

## Abstract

**Graphical Abstract:**
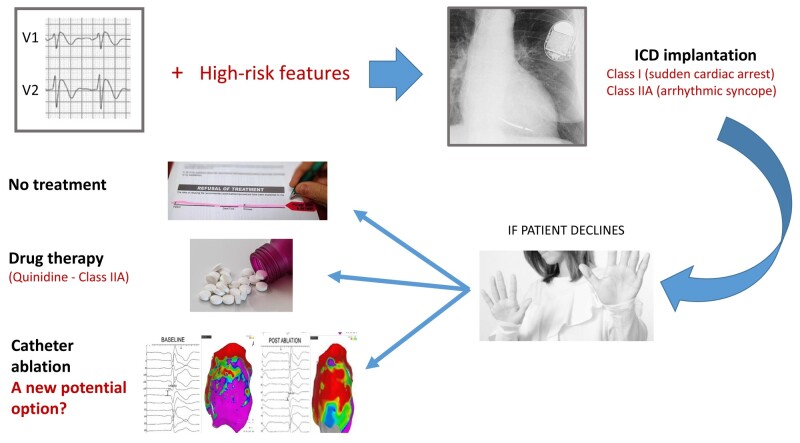

A patient’s implantable cardioverter-defibrillator (ICD) refusal poses a relevant therapeutic challenge for cardiologists managing patients with Brugada syndrome (BrS) and a clear indication to ICD implantation for primary or secondary prevention.

Despite current ESC guidelines recommendations, ICD implantation may not be considered straightforward by patients with BrS that may refuse for different reasons, a globally accepted therapeutic strategy to prevent sudden cardiac death.^1–5^

As alternative option, guidelines indicate to consider quinidine in patients who qualify for an ICD but have a contraindication or decline this intervention.^4^ However, adverse effects of quinidine can occur in up to 37% of patients, and the drug is inaccessible in many countries, including those where the syndrome is considered endemic.^6–8^ Other non-pharmacological strategies have not been investigated nor proposed so far.

Li *et al.*^5^ report for the first time the outcomes of catheter ablation (CA) in a specific population of high-risk patients with spontaneous BrS declining ICD implantation. In their retrospective study, 40 patients were treated with ICD implantation (*n* = 22) or CA only (*n* = 18). Study population presented with both spontaneous Type 1 Brugada electrocardiogram (ECG) and symptoms (cardiac arrest survivors or syncope of arrhythmic origin). The high-risk profile of patients was reflected by a mean Shanghai score of 7.

An interesting finding of the study concerned the age of patients treated with CA. The CA group was significantly younger compared with the ICD group (39 ± 9 vs. 48 ± 8, *P* = 0.02), which was attributed by the authors to a higher likelihood of younger patients declining device implantation. The potential reasons for refusing an ICD are multiple, ranging from the perceived high risk of procedural complications to the psychological burden following the diagnosis and further indications to invasive procedures. Indeed, ICD therapy is related to a non-negligible rate of device-related issues highly affecting young patients’ quality of life.^9^

Moreover, new-onset depression or anxiety following a diagnosis of BrS is a recognized condition affecting up to one-sixth of patients with BrS.^10^ In addition, it has been recently reported that mental distress and Type D personality are significantly more common in BrS patients compared with the general population.^11^ This clearly highlights the importance of including mental health screening and care as standard for BrS and supporting psychological patients qualifying for an ICD implantation.

In this study, the strategy adopted for patients refusing an ICD was endo-epicardial mapping and ablation of the arrhythmogenic substrate in the majority of cases (83%) followed by ventricular fibrillation (VF)-triggering premature ventricular complexes (PVCs) ablation (17%). Although, ajmaline is considered the best drug agent for electro-anatomic mapping of the arrhythmogenic substrate, in this study intravenous administration of propafenone was used. The unavailability of ajmaline is a recognized issue when performing the diagnostic assessment of patients with suspected BrS and epicardial mapping procedures in many countries. The epicardial arrhythmogenic substrate and PVCs triggering VF are two recognized ablation targets in high-risk patients with BrS and have been successfully used to reduce ICD shocks recurrences refractory to drug therapy.^12,13^ Moreover, the abolition of epicardial arrhythmogenic substrate, characterized by prolonged fragmented ventricular potentials, has been associated to ECG pattern normalization and ventricular tachycardia (VT)/VF non-inducibility at programmed ventricular stimulation.^13^ The role of triggers from the Purkinje arborization or the right ventricular outflow tract in initiating ventricular fibrillation associated with BrS has been previously reported.^14^ Despite this evidence, CA has not been tested so far as a therapeutic alternative in patient refusing ICD implantation.

In this study, a long-term efficacy of CA was shown. During a median follow-up of 46 months, CA proved to be efficient as an arrhythmic event-free survival was observed in 94% of patients treated with CA and in 45% of ICD carriers.

Although the relatively small sample size of the study may limit the generalizability of the results, the use of non-pharmacological strategies to protect patient refusing an ICD is promising and deserves further attention in the near future.
